# Cross-bridge model-based quantification of muscle metabolite alterations leading to fatigue during all-out knee extension exercise

**DOI:** 10.3389/fphys.2026.1741796

**Published:** 2026-03-20

**Authors:** John I. Hendry, Muhammet Enes Erol, Gwenael Layec, Edward P. Debold, Anders Wallqvist, Venkat R. Pannala

**Affiliations:** 1 Department of Defense Biotechnology High Performance Computing Software Applications Institute, Defense Health Agency Research & Development, Medical Research and Development Command, Fort Detrick, MD, United States; 2 The Henry M. Jackson Foundation for the Advancement of Military Medicine, Inc., Bethesda, MD, United States; 3 Department of Kinesiology, University of Massachusetts, Amherst, MA, United States; 4 School of Health and Kinesiology, University of Nebraska, Omaha, NE, United States

**Keywords:** cross-bridge cycle, inorganic phosphate, knee extension, protons, skeletal muscle fatigue

## Abstract

Intense physical exercise is associated with high energy demands and muscle metabolite changes that affect force generation, leading to muscle fatigue. Although these changes are well characterized in humans, their contribution to muscle fatigue is not clearly understood. Furthermore, we lack experimental methodologies for a systems-level exploration of these changes that occur during intense exercise to understand the mechanisms behind muscle fatigue development. In this study, we updated our previously developed human skeletal muscle model to include new proton-binding mechanisms and adapted it to study fatigue development during an intense all-out knee extension exercise. We contextualized and parameterized the updated model to simulate muscle force generation and muscle metabolite alterations, using motor unit recruitment data obtained from human subjects performing an all-out knee extension exercise. Our model predictions showed that nullifying the observed decline in motor unit recruitment during all-out exercise was not sufficient to stop fatigue development, as the force recovered only by 13%, and suggested that other factors may play a role. We found that the accumulation of inorganic phosphate (P_i_) and protons (H^+^), both individually (P_i_ by ∼9% and H^+^ by ∼31%) and synergistically (∼42%), were the main contributing factors at the cross-bridge level that inhibited force generation during all-out exercise. Our model simulations showed that force generation was more sensitive to H^+^ than P_i_ during an all-out knee extension exercise, with elevated P_i_ levels promoting actin-myosin detachment and elevated H^+^ levels preventing the formation of strongly bound cross-bridge states. Furthermore, our computational analysis revealed that the accumulation of H^+^ during an all-out knee extension exercise is the key contributing factor responsible for fatigue development as compared to P_i_ during a constant-power plantar flexion exercise.

## 1 Introduction

Intense muscle contraction that leads to muscle fatigue is accompanied by drastic biochemical and metabolic changes in contracting muscle fibers (Tesch et al., 1986; Sahlin and Ren, 1989; Spriet et al., 1989; Kim et al., 1995; Sundberg and Fitts, 2019). The decline in muscular force generation brought about by these biochemical changes, referred to as peripheral fatigue (Allen et al., 2008), has been the focus of scientific investigations for decades. This peripheral fatigue phenomenon is distinct from central fatigue, which is characterized by a central nervous system-induced decrease in motor unit recruitment that negatively impacts muscular force output (Gandevia, 2001). The actin-myosin cross-bridge cycle that lies at the heart of muscle contraction and force generation is powered by adenosine triphosphate (ATP) hydrolysis. Therefore, muscle contraction is accompanied by an accumulation of ATP hydrolysis products, such as adenosine diphosphate (ADP), inorganic phosphate (P_i_), and protons (H^+^) (Zewe and Fromm, 1962; Gevers, 1977; Zilva, 1978; Wilkie, 1979; Tesch et al., 1986; Sahlin and Ren, 1989; Spriet et al., 1989; Dennis et al., 1991; Kim et al., 1995), and activation of creatine kinase, which buffers ATP levels by regenerating ATP using the intracellular stores of phosphocreatine (PCr), resulting in a rapid decrease in cellular PCr content (Wyss and Kaddurah-Daouk, 2000). Furthermore, depending on exercise intensity and oxygen supply, glycolysis and oxidative phosphorylation interact to resupply ATP, resulting in further biochemical changes (Gastin, 2001).

Previous studies have characterized the metabolic changes associated with intense, fatiguing, contractile activity in humans using ^31^P magnetic resonance spectroscopy (^31^P-MRS) (Vanhatalo et al., 2011; Broxterman et al., 2017). These studies reported that during intense fatiguing exercise, intracellular P_i_ increases from a resting state level of 3–5 mM to >30 mM (Kemp et al., 2007; Broxterman et al., 2017); pH decreases from 7.0-7.1 to as low as 6.2 (Wilson et al., 1988; Cady et al., 1989; Broxterman et al., 2017); ADP increases from ∼5–10 μM to 200 μM (Cooke, 2007); and PCr decreases from ∼35 mM to ∼5 mM (Broxterman et al., 2017). However, how and to what extent these metabolite alterations impact muscle force generation in exercising humans is not yet fully characterized. *In vitro* studies using muscle fibers isolated from rats, rabbits, and humans have shown that elevated P_i_ and H^+^ concentrations impact muscle force generation both individually (Pate and Cooke, 1989; Coupland et al., 2001; Debold et al., 2004; Knuth et al., 2006; Sundberg et al., 2018) and synergistically (Karatzaferi et al., 2008; Nelson et al., 2014); however, these experimental studies do not fully recapitulate the *in vivo* conditions of the muscles when humans perform intense fatiguing exercise. In humans, skeletal muscle force production does not occur as a single homogeneous process but emerges from the dynamic recruitment and discharge rate modulation of individual motor units, each comprising muscle fibers with distinct force–velocity characteristics.

A systematic measurement and analysis of complementary human muscle physiology data, such as metabolite changes, force output, and motor unit recruitment, collected under the same fatiguing exercise conditions would help address the above knowledge gap. While noninvasive techniques, such as surface electromyography (EMG) (Sun et al., 2022; Pitzalis et al., 2025) and ^31^P-MRS (Shenton et al., 1986; Kemp et al., 2007; Vanhatalo et al., 2010; Sundberg et al., 2019; Bartlett et al., 2020; Fitzgerald et al., 2023), combined with exercise protocols that focus on specific muscle types (e.g., knee extension) have provided the simultaneous measurement of the necessary data, we still lack a framework to analyze the data in an integrated fashion. For example, several previous computational studies employed experimental data from isolated human and animal muscle fibers to address different aspects of muscle physiology independently, such as actin-myosin cross-bridge kinetics (Walcott et al., 2012; Herzog and Schappacher-Tilp, 2023), skeletal muscle metabolism (Lambeth and Kushmerick, 2002; Lai et al., 2008; Lopez et al., 2020), and metabolite-mediated inhibition of cross-bridge kinetics (Pate and Cooke, 1989; Tewari et al., 2016), but these models need to be extended or combined into one framework that facilitates the integrated analysis of diverse human muscle physiological data collected under the same fatiguing exercise condition. Given that our understanding of muscle physiology during exercise is limited by methods and human research ethics, such computational models are necessary to expand on our current knowledge by simulating conditions that we cannot study using experimental techniques.

In a recent study, we developed a combined framework that accounts for cross-bridge kinetics, muscle metabolism, metabolite-mediated inhibition, and motor unit recruitment and performed an integrated analysis of data collected from humans performing a constant-work-rate plantar flexion exercise (Hendry et al., 2025). Our analysis indicated that P_i_ plays a major role in muscle fatigue development while H^+^ accumulation has only a marginal effect. However, given that muscle fatigue development significantly depends on the exercise type, intensity, and duration as well as the muscle groups involved (e.g., vastus lateralis, gastrocnemius, etc.), we need further studies using computational tools to fully understand muscle force generation during different exercise modalities and to identify potential contributing factors that lead to muscle fatigue. For example, compared to the low-intensity, constant-work-rate plantar flexion exercise performed in our previous study, an all-out knee extension exercise involves a maximum effort, a progressive decline in force generation, and the use of a single muscle group (i.e., the quadriceps) (Andersen et al., 1985; Taylor and Gandevia, 2008).

In this study, we adapted and updated our earlier human skeletal muscle model (Hendry et al., 2025) to analyze experimental data collected during an all-out knee extension exercise to understand the underlying mechanisms and identify potential metabolic factors contributing to muscle fatigue. From the literature, we obtained human physiological data that included integrated muscle force measurements, motor unit recruitment profiles (integrated EMG, iEMG), and changes in the levels of H^+^, P_i_, and PCr during an all-out knee extension exercise (Burnley, 2009; Austin et al., 2010; Brennan et al., 2017; Broxterman et al., 2017). We used these datasets to parameterize the cross-bridge model and adapted it to simulate the force generation and metabolite levels in the quadriceps muscles during this exercise modality. The model simulated force generation and metabolite changes by accounting for actin-myosin cross-bridge cycling and energy shuttles or pathways. We used the parametrized model to study the effect of motor unit recruitment, H^+^, and P_i_ on muscle force generation and fatigue development during an all-out knee extension exercise and compared our findings with those for a constant-power plantar flexion exercise. We hypothesized that the observed progressive decrease in motor unit recruitment and alterations in muscle metabolite pools of P_i_ and H^+^ impact force generation during all-out knee-extension exercise and represent potential key contributing factors to fatigue development.

## 2 Materials and methods

### 2.1 Experimental data for model parameterization

For model parameterization, we compiled literature data, including integrated force, intracellular metabolite levels, and motor unit recruitment (iEMG), that were recorded in human quadriceps muscles during an all-out knee extension exercise. We obtained the integrated force and intracellular metabolite data from the study of Broxterman et al. (2017). Briefly, eight healthy men (mean ± SD: age 25 ± 5 years, height 178 ± 4 cm, weight 78 ± 8 kg) performed 60 single-leg, maximal voluntary isometric quadriceps contractions (3-s contraction and 2-s relaxation) in 5 min while inside a whole-body magnetic resonance imaging (MRI) system. Subjects performed the exercise in a semi-recumbent position, with the knee of the exercising leg supported by a knee support (∼45° knee angle), the ankle attached to an immovable strain gauge, and the hips and thigh held in position using non-elastic straps. The exercise invoked near-maximal voluntary motor unit recruitment in the quadriceps. The force generated during the exercise was measured (sample interval: 5 s) by the immovable strain gauge (SSM-AJ-250, Interface) attached to the ankle. The ^31^P-MRS data corresponding to metabolite alterations were acquired using a 2.9-T MRI system (Tim-Trio, Siemens Medical Systems, Munich, Germany) operating at 49.9 MHz (^31^P resonance) and a dual-tuned ^31^P-^1^H surface coil with linear polarization. We obtained the motor unit recruitment pattern in the form of iEMG data from the study of Burnley (2009), where eight healthy men (mean ± SD: age 29 ± 6 years, height 178 ± 9 cm, weight 77.3 ± 11.3 kg) performed 60 maximal voluntary isometric quadriceps contractions (3-s contraction and 2-s relaxation) in 5 min. The study used bipolar Ag-AgCl electrodes to sample the EMG of the vastus lateralis at 1 kHz. The raw signals were amplified using a bio-amplifier (gain 1000) and filtered using a third-order Butterworth filter.

### 2.2 Modeling skeletal muscle force generation using cross-bridge cycling

**FIGURE 1:**
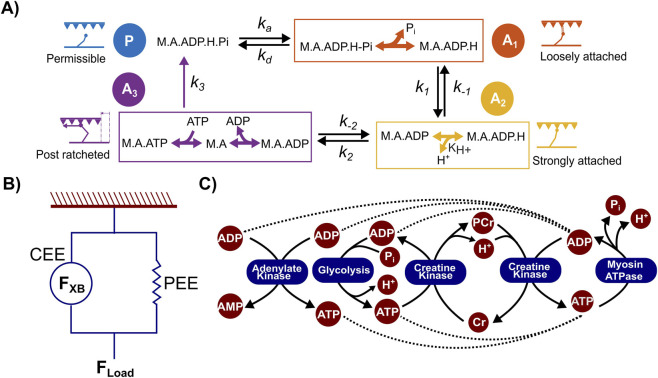
A 4-state cross-bridge model to simulate all-out knee extension exercise. **(A)** Schematic illustrating the modified cross-bridge model used to simulate force generation. P represents the permissible state, wherein actin (A) and myosin (M) have not initiated any interaction but are free to do so without being blocked by troponin. A_1_ represents the state in which myosin is loosely bound to actin, A_2_ denotes the pre-power stroke state featuring a strong binding between actin and myosin, and A_3_ represents the post-power stroke state, where myosin is still bound to actin. *k*_*a*_ *, k*_*d*_ *, k*_*1*_ *, k*_*-1*_ *, k*_*2*_ *, k*_*-2*_, and *k*_*3*_ represent the rate constants that model the transitions between the above four states. Each of the bound states (A_1_, A_2_, and A_3_) is comprised of different actomyosin complexes that are in rapid equilibrium with each other (as shown in the boxes). As indicated, the transitions between some of these complexes require the association/dissociation of metabolites, such as ATP, ADP, H^+^, and P_i_. **(B)** The overall force generation scheme of the model. Total force (F_Load_) is the sum of the force generated by the parallel elastic element (PEE; F_PEE_) and the contractile element (CEE; F_XB_). **(C)** Schematic illustration of the metabolic pathways included in the model to account for the metabolite dynamics that accompany cross-bridge cycling. Cr, creatine; PCr, phosphocreatine.

To simulate muscle force generation and metabolite alterations, we used a modified version of our previously developed cross-bridge cycling model (Hendry et al., 2025). To better capture the motor unit recruitment profile characterizing all-out exercise, we assumed that actomyosin cross-bridge formation was activated to the extent determined by the iEMG value measured during an all-out knee extension exercise, with all the muscles fully activated at the start of the exercise. Therefore, we modified our previous model so that the fraction of the actin and myosin in the permissible state (P) (actin and myosin detached but free to bind to each other) was directly determined by the iEMG value (Equation 1). This modification rendered the actin-myosin non-permissible state (N) (actin sites blocked by troponin preventing the formation of actomyosin complexes), which was part of our earlier model (Hendry et al., 2025), redundant, resulting in the four-state cross-bridge model shown in Figure 1. We also assumed that the ATP-dependent last step in the cross-bridge cycle (A_3_ state) resulted in the detachment of actin and myosin (permissible state P) along with the bound ATP hydrolysis products (ADP, P_i_, and H^+^) attached to this state (illustrated in Figure 1A). Therefore, we adopted the sequential release of ATP hydrolysis products as a potential mechanism for the subsequent cross-bridge state transitions that lead to force generation (Tran et al., 2010). According to this theory, release of P_i_ from the permissible state (P) in a rapid equilibrium step results in a loosely attached cross-bridge cycling state (A_1_), followed by a proton-release step that yields the strongly attached cross-bridge state (A_2_) (Sundberg et al., 2018), which is a prerequisite for the release of ADP in the post-ratcheted force-generating state (A_3_). Thus, the H^+^ formed as a byproduct of ATP hydrolysis (A_3_→P transition) or from other potential sources (Figure 1C) remains attached to the actomyosin complex and is released during the A_1_→A_2_ transition of the subsequent cross-bridge cycle. We provide the complete list of model equations governing the dynamics of the cross-bridge states (P, A_1_, A_2_, and A_3_) as well as the metabolite interactions and their relation to force production in Supplementary Appendix A in the Supplementary Material. We calculated the overall force as the sum of the force generated from cross-bridge cycling and the force from parallel elastic elements (Figure 1B). To simulate the dynamics of P_i_, H^+^, and PCr, we modeled the following metabolic processes using mass action kinetics: *1*) myosin-associated ATP hydrolysis (Lymn and Taylor, 1971), *2*) creatine kinase (Meyer et al., 1984), *3*) ATP-generating processes (Gastin, 2001), and *4*) adenylate kinase (Janssen et al., 2003), as illustrated in Figure 1C. We provide the corresponding set of differential equations that govern the dynamics of all metabolites implicated in these processes (ATP, ADP, PCr, P_i_, and H^+^) in Supplementary Appendix B in the Supplementary Material.

### 2.3 Simulating knee extension cycles

To simulate a single knee extension cycle, we simultaneously solved Supplementary Appendix Equations A1–A10 in Appendix A and Supplementary Appendix Equations B1–B5 in Appendix B for a cycle period (*T* ^*cyc*^) determined by the experimental protocol to evaluate the time-dependent metabolite levels and fractions of different cross-bridge states. We initiated each cycle by setting the fractions A_1_, A_2_, and A_3_ to zero and using the following equation to initiate the P state:

$${P\left(t_{0}\right)}^{j}=\text{iEMG}\left(j\right)$$ (1)

where *j* represents the index of the current cycle and *t*_0_ denotes the beginning of the cycle. For the metabolites, we set their initial levels for the first cycle to the levels estimated before the start of the exercise (*t* = 0). For the subsequent cycles, we initiated the metabolite levels from their final concentrations in the previous cycle, allowing for accumulation. We implemented and simulated the model in the MATLAB (R2022a) environment. The models used in this study are publicly available at GitHub (https://github.com/BHSAI/Dynamic_knee_extension_model).

### 2.4 Model parameterization

To estimate the model parameters, we used a nonlinear least-squares method to fit the model-simulated and experimental data for force (F_total_) and intracellular levels of PCr, P_i_, and H^+^ for 60 knee extension cycles (*N*_*c*_). We used the following nonlinear least-squares objective function (*f*_*obj*_) (Equation 2) for our parameterization routine:

$$\begin{array}{ll} & f_{obj}=\frac{\sum_{j=1}^{N_{c}}{\left({\left[P_{i}\right]}_{j}^{\text{data}}‐{\left[P_{i}\right]}_{j}^{\text{Model}}\right)}^{2}}{\max\left({\left[P_{i}\right]}_{j}^{\text{data}}\right)}\\ & \;+\frac{\sum_{j=1}^{N_{c}}{\left({\left[H^{+}\right]}_{j}^{\text{data}}‐{\left[H^{+}\right]}_{j}^{\text{Model}}\right)}^{2}}{\max\left({\left[H^{+}\right]}_{j}^{\text{data}}\right)}\\ & \;+\frac{\sum_{j=1}^{N_{c}}{\left({\left[\text{PCr}\right]}_{j}^{\text{data}}‐{\left[\text{PCr}\right]}_{j}^{\text{Model}}\right)}^{2}}{\max\left({\left[\text{PCr}\right]}_{j}^{\text{data}}\right)}+\frac{\sum_{j=1}^{N_{c}}{\left({F_{\text{total}}}_{j}^{\text{data}}‐{F_{\text{total}}}_{j}^{\text{Model}}\right)}^{2}}{\max\left({F_{\text{total}}}_{j}^{\text{data}}\right)} \end{array}$$ (2)

We used the MATLAB “fmincon” function to minimize the above objective function and estimate the parameters. We repeated the parameter estimation 100 times starting from random points within the sample space. We chose the parameter set with *f*_*obj*_ closest to zero as the best-fit parameter set.

### 2.5 Sensitivity analysis

After identifying the best-fit parameter set for the model, we evaluated the model’s sensitivity to different parameters using both local and global sensitivity analyses. For the local sensitivity analysis, we perturbed the parameters by 1%, one at a time, and calculated the relative change in force (Wei et al., 2007; Nagaraja et al., 2014). We used Equation 3 to calculate the sensitivities:

$$S_{i\left(x^{0}\right)}=\frac{\frac{F_{\text{total}}\left(x_{1}^{0},\ldots ,x_{i}^{0}+dx_{i},\ldots ,{\;x}_{I}^{0}\right)-F_{\text{total}}\left(x_{1}^{0},\ldots ,x_{i}^{0}-dx_{i},\ldots ,x_{I}^{0}\right)}{F_{\text{total}}\left(x^{0}\right)}}{\left(2\times \frac{dx_{i}}{x_{i}^{0}}\right)}$$ (3)

where $S_{i}$ represents the model sensitivity with respect to the *i*th parameter, $x^{0}$ denotes the best-fit parameter set, and dx represents the change in the parameter. For the global sensitivity analysis, we uniformly sampled the 10% neighborhood of the best-fit parameter set (*x* ^0^) to obtain 10,000 parameter sets (Wei et al., 2007; Nagaraja et al., 2014). For uniform sampling of the neighborhood of $x^{0}$ , we used Latin hypercube sampling as implemented in MATLAB (function “lhsdesign”). For each of the 10,000 different models defined by the sampled parameter sets, we performed a local sensitivity analysis. We then used box plots to visualize and evaluate the parameter sensitivities calculated from these 10,000 models. The modeling results presented herein were independently assessed for reproducibility.

## 3 Results

### 3.1 Characterizing skeletal muscle force-generation dynamics during an all-out knee extension exercise

**FIGURE 2:**
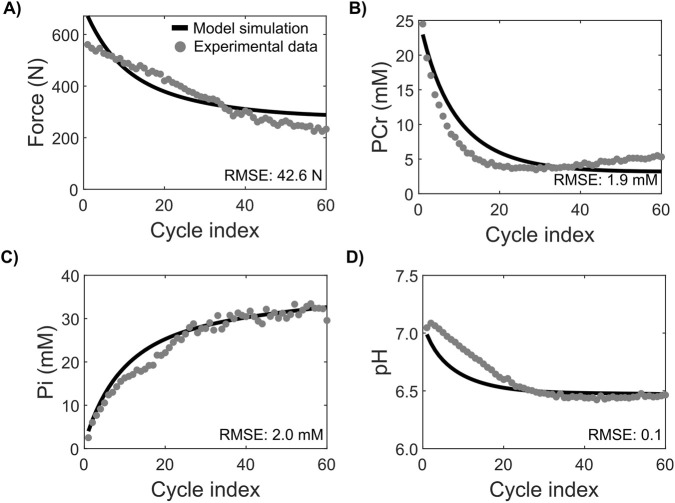
Cross-bridge model parameterized to simulate force generation characteristics during all-out knee extension exercise. **(A)** Plot comparing the model-simulated force (continuous line) with the experimentally measured force (gray dots) reported in Broxterman et al. (2017). **(B–D)** Plots comparing the model-simulated alterations in intramuscular metabolite levels with the experimental data for phosphocreatine (PCr) **(B)**, P_i_ **(C)**, and pH **(D)** reported in Broxterman et al. (2017). RMSE, root mean square error.

We adapted our previously developed cross-bridge model for plantar flexion exercise (Hendry et al., 2025) to simulate all-out knee extension cycles and further updated the model formulation such that H^+^ ions generated during ATP hydrolysis remain attached to the actomyosin complex until they are dissociated at the A_2_ state. Thus, based on these modifications, the rates of both the A_1_→A_2_ and A_2_→A_3_ transitions during cross-bridge cycling were impacted by H^+^ concentration, requiring re-estimation of all the kinetic parameters in the model. For the model to accurately simulate force generation and metabolite alterations during an all-out knee extension exercise, we needed to estimate 24 parameters (Table 1) using previously reported experimental data. We used the iEMG data from Burnley (2009) as input to the model (as described in Section 2.3) and fitted the model’s simulations to force, PCr, P_i_, and H^+^ data from Broxterman et al. (2017) (as detailed in Section 2.4) to estimate the various parameters related to cross-bridge cycling, such as the rate constants for cross-bridge state transition, dissociation constants governing the association/dissociation of various metabolic factors with cross-bridge complexes, stiffness constants for attached and post-ratcheted states, stretch-sensing parameters, and rate constants for various metabolic processes. Figure 2 compares the model simulations for the best-fit parameter set against the experimental data for force, PCr, P_i_, and H^+^ and displays the associated root mean square error (RMSE) values. Our parameterization procedure matched the force data with an RMSE value of 42.6 N (Figure 2A). Similarly, we fitted PCr (Figure 2B) and P_i_ (Figure 2C) data with RMSE values of 1.9 mM and 2.0 mM, respectively. For pH, we obtained a fit with an RMSE value of 0.1 pH units (Figure 2D). Thus, our model successfully recapitulated the reported dynamics of force generation, PCr, P_i_, and H^+^ in quadriceps muscle during an all-out knee extension exercise.

**TABLE 1:** Estimates of model parameters for an all-out knee extension exercise compared to those for a dynamic plantar flexion exercise.

Parameter	Description	Knee extension	Plantar flexion^a^	Units
Cross-bridge cycle parameters				
*k*_*a*_	Rate of actin-myosin transition from permissible state to loosely attached state	1305.6	313.1	s^-1^
*k*_*d*_	Rate of actin-myosin transition from loosely attached state to permissible state	1482	59	s^-1^
*k*_*1*_	Rate of cross-bridge transition from loosely bound to strongly bound state	1419.4	7.5	s^-1^
*k*_*-1*_	Rate of cross-bridge transition from strongly bound to loosely bound state	1004	74	s^-1^
*k*_*2*_	Rate of ratcheting	638	104.2	s^-1^
*k*_*-2*_	Rate of unratcheting	1077	133	s^-1^
*k*_*3*_	Rate of actin-myosin detachment	371.2	183.4	s^-1^
*α*_*1*_	Stretch sensing parameter for *k*_*1*_ and *k*_*-1*_	74.2	1.0	μm^-1^
*α*_*2*_	Stretch sensing parameter for *k*_*2*_	405	143.8	μm^-1^
*α*_*3*_	Stretch sensing parameter for *k*_*3*_	40.4	40	μm^-1^
*s*_*3*_	Stretch in state A_3_ at which *k*_*3*_ is minimum	0.1	98.2	nm
*K*_*ATP*_	ATP dissociation constant	3.3	2.7 × 10^−1^	mM
*K*_*ADP*_	ADP dissociation constant	18.6 × 10^−5^	3.9 × 10^−5^	mM
*K*_*Pi*_	P_i_ dissociation constant	40.9	3	mM
*K*_*H+*_	H^+^ dissociation constant	6.2 × 10^−1^	5.8 × 10^−1^	μM
Force generation parameters				
*k*_*stiff,1*_	Stiffness constant of frictional forces during actin-myosin interaction	265.8	72,013.2	mN mm^-2^ μm^-1^
*k*_*stiff,2*_	Stiffness constant of forces generated during the cross-bridge power stroke	95,115.2	15,157.3	mN mm^-2^ μm^-1^
Metabolic parameters				
*k*_*CKf*_	Rate constant for creatine kinase (ATP forming)	7.5 × 10^−1^	0.5	mM^-1^ s^-1^
*k*_*CKr*_	Rate constant for creatine kinase (ADP forming)	0.3 × 10^−3^	3.0 × 10^−3^	mM^-1^ s^-1^
*k*_*Gly*_	Rate constant for ATP generation	9.3 × 10^−1^	2.4 × 10^−1^	mM^-1^ s^-1^
*k*_*Pi,dil*_	Rate constant for P_i_ dilution or export from myocytes	0.2 × 10^−3^	3.2 × 10^−3^	s^-1^
*k*_*adk*_	Rate constant for adenylate kinase	55.3	18.1	mM^-1^ s^-1^

^a^ Plantar flexion exercise parameters are as reported in Hendry et al. (2025).

Table 1 shows the model parameters estimated for the all-out knee extension exercise in this study compared to those for the constant-power plantar flexion exercise used in our earlier study (Hendry et al., 2025). The parameter estimates differ between these two studies, at least by an order of magnitude in certain cases, and most of these differences can be explained by the type of experimental exercise and muscles involved. For example, the all-out knee extension exercise involved maximal voluntary contractions for 5 min (Broxterman et al., 2017), while the plantar flexion exercise involved constant-power, submaximal contractions above critical power for 10 min. The knee extension exercise predominantly engaged the quadriceps muscle, while the plantar flexion exercise engaged the gastrocnemius muscle. The magnitude of the force generated in the all-out knee extension exercise was at least 550 N, while the constant-power plantar flexion exercise maintained the force output at around ∼54 N. These differences highlight that the temporal dynamics of muscle metabolite alterations significantly differed between the exercise types. For example, the rates of P_i_ and H^+^ accumulation in the all-out knee extension exercise were much higher than in the constant-power plantar flexion exercise. In the first 1 min, P_i_ levels increased 16-fold during the all-out knee extension exercise (Figure 2C) as opposed to 4-fold during the constant-load plantar flexion exercise. Similarly, in the first 2 min, H^+^ levels increased 300% during the all-out knee extension exercise (Figure 2D) compared to 31% during the plantar flexion exercise. We observed a similar behavior for the rate of PCr consumption, where PCr levels decreased within the first 1 min by 77% during the all-out knee extension exercise (Figure 2B) compared to 43% during the constant-load plantar flexion exercise. In addition, by the end of the exercise, we observed a much higher accumulation of H^+^ during the all-out knee extension compared to the constant-load plantar flexion exercise, indicating that exercise modality largely determines the muscle metabolite factors that contribute to fatigue development. Further illustrating the role of exercise type, the rate parameters associated with cross-bridge kinetics under the all-out knee extension exercise were much higher than those for the constant-load plantar flexion exercise, indicating faster cross-bridge kinetics under the knee extension exercise. We attribute this to a much quicker temporal variation in muscle metabolites (P_i_, H^+^, and PCr) that are closely tied to ATP consumption by the cross-bridge cycle (Barclay, 2017; Hargreaves and Spriet, 2020).

### 3.2 Effect of motor unit recruitment on force generation during an all-out knee extension exercise

**FIGURE 3:**
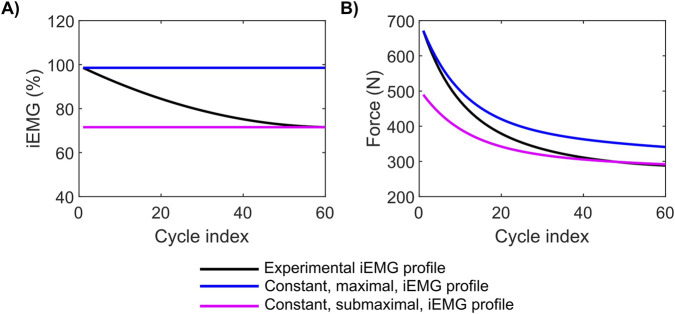
Effect of motor unit recruitment on force generation capacity during an all-out knee extension exercise. **(A)** Plot showing the experimentally recorded motor unit recruitment (iEMG) profile (black line) during an all-out knee extension exercise (Burnley, 2009) and the hypothetical profiles corresponding to constant maximal motor unit recruitment (blue line) and constant submaximal activation levels equal to the value recorded in the final cycle (magenta line). **(B)** Plot comparing the force generation profiles for the recorded (black) and hypothetical profiles (blue line and magenta line).

Experimental observations have shown that motor unit recruitment, measured by iEMG, progressively decreased as all-out exercise progressed (Vandewalle et al., 1991; Gandevia et al., 1996; Burnley, 2009). For example, Burnley reported an ∼30% decline in activation by the end of the exercise in their study (Burnley, 2009). We used our updated model to investigate how this characteristic decline in motor unit recruitment affects force generation during all-out knee extension exercise. Specifically, we aimed to determine to what extent the observed decrease in motor unit recruitment contributes to the observed decline in force generation during an all-out knee extension exercise. To address this question, we investigated the model’s force generation under two different iEMG profiles (Figure 3A): the iEMG profile reported by Burnley for an all-out knee extension exercise (Burnley, 2009) (black line) and an iEMG profile that we assumed to remain constant at the initial value throughout the entire exercise, without any decrease (blue line). Figure 3B shows the force generation profiles for these two different motor unit recruitment profiles. Our simulations showed that the constant maximal motor unit recruitment (100%) profile was able to marginally recover the force-generating capacity (Figure 3B, blue line), with an ∼52 N smaller decrease in force generation at the end of 60 cycles compared to the original trend (Figure 3B, black line). This corresponds to a 13% recovery of the drop in force observed for the experimental profile. Thus, our results showed that even during a hypothetical scenario where subjects provided full effort to maintain maximal motor unit recruitment level, they were not able to fully recover force production during the course of the all-out knee extension exercise, indicating that additional factors potentially contribute to the observed reduction in force-generating capacity. In comparison, if we maintained motor unit recruitment at submaximal levels (70%), as observed at the end of the exercise (Figure 3A, magenta line), force production followed a similar trajectory of decline, but with a significant drop in the subject’s capacity to produce the required force at the beginning of the exercise itself (Figure 3B, magenta line).

### 3.3 Effect of P_i_ and H^+^ accumulation on force generation during an all-out knee extension exercise

**FIGURE 4:**
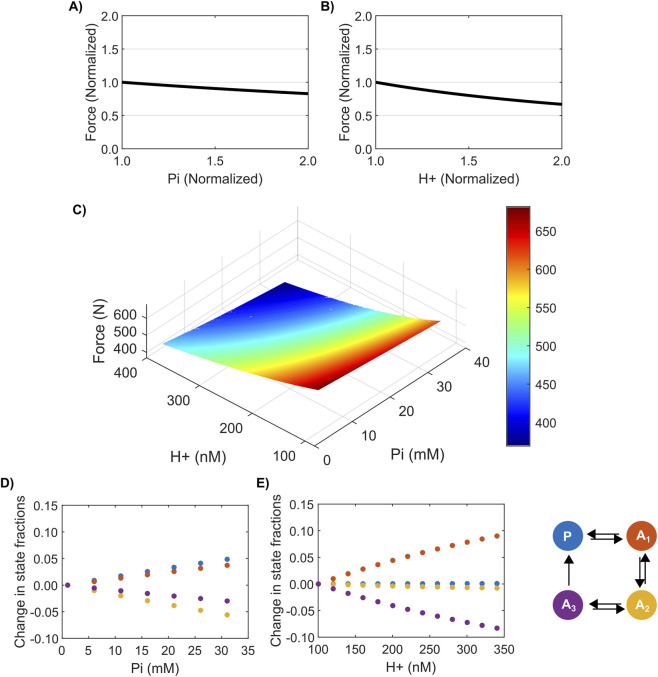
Effect of P_i_ and H^+^ on force generation. **(A,B)** Plots showing the effect of P_i_ **(A)** and H^+^ **(B)** on normalized force during an all-out knee extension exercise. The y-axes represent the force generated at the end of 60 + 6 cycles, normalized by force at the end of 60 cycles. The x-axes show the concentration of P_i_/H^+^ normalized by its respective concentration at the end of 60 cycles. **(C)** Surface plot showing the effect of a simultaneous change in H^+^ and P_i_ on force generation. **(D,E)** Plots showing the effect of accumulation of P_i_ **(D)** and H^+^ **(E)** on the fraction of different cross-bridge states.

To quantify other potential factors leading to the decline in muscle force generation during all-out exercise, we studied the effect of intracellular P_i_ and H^+^ levels because these metabolic factors have been implicated in inhibiting muscle force generation (Pate and Cooke, 1989; Coupland et al., 2001; Debold et al., 2004; Knuth et al., 2006; Sundberg et al., 2018). First, we simulated 60 knee extension cycles as described in Section 2.3. Then, we simulated six additional cycles (10% of the total cycles) under further elevated concentrations of P_i_ or H^+^, ranging from one to two times the concentration at the end of 60 cycles. When simulating the last six cycles, we set the rate of change for the concentration of all other metabolites to zero. We observed that both P_i_ (Figure 4A) and H^+^ (Figure 4B) negatively impacted muscle force generation. For a twofold increase in the concentration of P_i_, our model predicted an ∼17% decrease in force generation. Similarly, a twofold increase in H^+^ concentration decreased the force by ∼33%. Thus, increasing the H^+^ concentration had a greater impact on reducing force generation than increasing the P_i_ concentration.

In the above simulations, we varied H^+^ and P_i_ independent of each other to discover their individual effects. However, we also wanted to evaluate the effect of varying them simultaneously. To this end, we constructed a 100 × 100 grid of H^+^ and P_i_ level combinations that uniformly covered the entire range of H^+^ and P_i_ concentrations observed from the beginning to the end of the exercise protocol. We simulated one knee extension cycle under each H^+^ and P_i_ combination in the grid and visualized the alterations in force generation in a three-dimensional surface plot (Figure 4C). Our results clearly illustrate that force decreased faster with the increasing H^+^ compared to P_i_ concentrations observed during an all-out knee extension exercise. Furthermore, our model predictions clearly showed a much-reduced force generation when both H^+^ and P_i_ levels were maximum, indicating a combined role for both of these factors in modulating skeletal muscle force generation during all-out knee extension exercise.

To further understand the mechanism of how H^+^ and P_i_ impact force generation, we studied the effect of increasing their concentrations on the four different cross-bridge state fractions. To this end, we simulated a single cycle of knee extension exercise at varying levels of P_i_ or H^+^ spanning the range of reported concentrations and calculated the changes in the final fractions of different cross-bridge states. We set the rate of change for all the other metabolites to zero during these simulations. Figure 4D shows the change in different cross-bridge state fractions for increasing P_i_ concentrations. With increasing P_i_ concentration, the fractions of the P and A_1_ states increased while the fractions of all the other states decreased, which clearly shows that increasing P_i_ slowed down the P→A_1_ and A_1_→A_2_ state transitions. Similarly, Figure 4E shows the effect of increasing H^+^ concentration on the different cross-bridge state fractions. With increasing H^+^ concentration, the fraction of the A_1_ state increased while the fractions of all the other states either decreased or remained the same, indicating that, like P_i_, increasing H^+^ concentration impacted the A_1_→A_2_ transition. These simulations suggest that increased concentrations of either P_i_ or H^+^ prevented the formation of strongly bound actin-myosin cross-bridges that are required for force generation.

### 3.4 Sensitivity analysis reveals that force generation is sensitive to the H^+^ and P_i_ dissociation steps in the cross-bridge cycle

**FIGURE 5:**
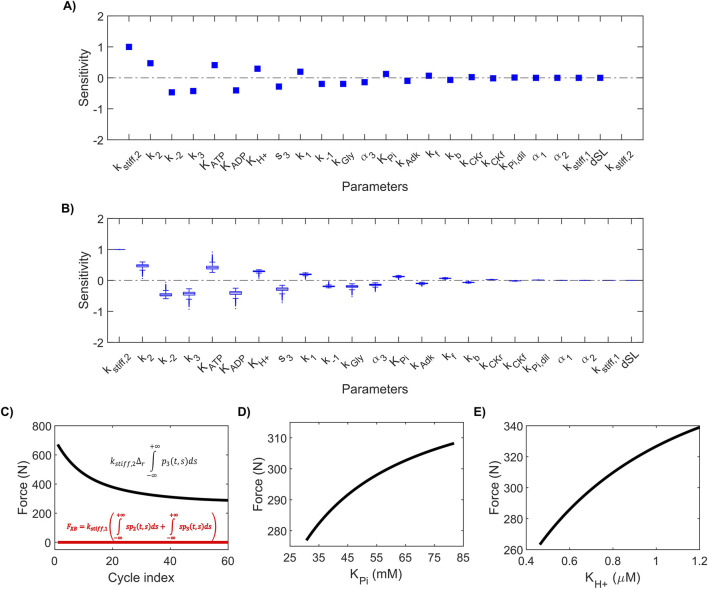
Parameter sensitivity analysis. **(A)** Plot of the local sensitivities calculated for all 24 parameters in the model. **(B)** Box plot showing the global sensitivities for all the parameters. **(C)** Plot showing the contribution of frictional forces arising from actin-myosin interaction [ $k_{stiff,1}\left(\int_{‐\infty }^{+\infty }{sp}_{2}\left(t,s\right)ds+\int_{‐\infty }^{+\infty }{sp}_{3}\left(t,s\right)ds\right)$ ] (red line) and forces generated during power stroke or ratcheting [ $k_{stiff,2}\Delta_{r}\int_{‐\infty }^{+\infty }p_{3}\left(t,s\right)ds$ ] (black line). **(D,E)** Plots showing how force varies as a function of the parameters *K*_Pi_ **(D)** and *K*_H+_ **(E)**.

Sensitivity analysis enables characterization of a model’s stability and consistency in the neighborhood of the best-fit parameter set. In addition, sensitivity values for individual parameters can provide deeper insight into the factors that affect muscle force generation. Therefore, we performed both local and global sensitivity analyses on the model parameters in simulating force generation. Figure 5A shows the sensitivity values calculated from the local sensitivity analysis, and Figure 5B compiles the sensitivity values calculated from the global sensitivity analysis in a box plot. Both the global and local sensitivity values ranged from −1 to 1, indicating that the model was stable during both local and global changes in parameter values. Furthermore, we found that the ranking of parameters based on the sensitivity analysis was conserved in both the local and global sensitivity analyses, indicating consistent model behavior during local and global changes in parameters.

Of the two stiffness constants (*k*_stiff,1_ and *k*_stiff,2_), the model’s force generation was more sensitive to *k*_stiff,2_, indicating a greater contribution of cross-bridge ratcheting to total force. Indeed, a closer investigation of the contribution from the two force-generating terms in Supplementary Appendix Equation A13 (Figure 5C) revealed that force was predominantly (∼100%) generated by the ratcheted cross-bridges. Similarly, between the dissociation constants for P_i_ (*K*_Pi_) and H^+^ (*K*_H+_), we found that force generation was more sensitive to *K*_H+_. To further confirm this, we simulated 60 knee extension cycles for a range of *K*_H+_ and *K*_Pi_ values varying between 0.75 and 2.00 times their best-fit value. Figure 5D and E show the final force at the end of the 60 cycles for these simulations, indicating that varying *K*_H+_ induced a larger change in force compared to that observed with a change in *K*_Pi_. Together, these results reveal that force generation is more sensitive to H^+^ than P_i_ during all-out knee extension exercise.

## 4 Discussion

In this study, we used a human skeletal muscle cross-bridge cycling model to understand how physiological factors, such as motor unit recruitment and metabolite alterations, contribute to the development of fatigue during intense exercise. We selected an all-out exercise modality as it involves maximal intensity and short duration, exhibits rapid accumulation of muscle metabolite pools, and results in a decline in the muscle’s ability to generate force, leading to fatigue development. Specifically, we aimed to elucidate the mechanisms behind fatigue development during an all-out knee extension exercise, as knee extension exclusively engages the quadriceps muscle with limited interference (e.g., force contribution and motor unit recruitment signals) from other adjacent muscles (Andersen et al., 1985), allowing noninvasive techniques, such as surface EMG (Sun et al., 2022) and ^31^P-MRS (Kemp et al., 2007), to identify exercise-induced physiological changes in the main contributor to force production, i.e., the quadriceps muscle (Taylor and Gandevia, 2008). To this end, we updated our previously developed human skeletal muscle model (Hendry et al., 2025) and adapted it to study all-out knee extension exercise. Briefly, the modified model accounts for a four-state cross-bridge cycle (Figure 1A) in combination with the key metabolic processes (Figure 1C) that orchestrate the metabolite alterations observed during intense exercise. In addition, we hypothesized that the H^+^ formed as a byproduct of ATP hydrolysis (A_3_→P transition) remains attached to the actomyosin complex and must be released as a prerequisite for the formation of a strongly bound state (A_2_) in the subsequent cross-bridge cycle. We formulated this hypothesis based on our previous work (Hendry et al., 2025), as well as information from other modeling studies (Tran et al., 2010), which showed that the proposed scheme captures the cross-bridge kinetics’ sensitivity to H^+^ concentration.

We used experimental data from multiple sources collected under similar all-out knee extension exercise protocols to inform and parameterize our model. For example, we used motor unit recruitment data in the form of iEMG [from Burnley (2009)] as input to the cross-bridge model and fitted the force and metabolite alteration data (PCr, H^+^, and P_i_) from Broxterman et al. (2017) to estimate the model parameters. The updated cross-bridge model successfully recapitulated the temporal evolution of force, PCr, H^+^, and P_i_ dynamics within the experimental error margin during a knee extension exercise (Figure 2). Both local and global sensitivity analyses indicated that the model is stable and consistent within the 10% neighborhood of the optimal parameter set (Figure 5). Overall, using diverse physiological data collected with different noninvasive techniques, we show that our computational model can be useful to perform an integrated analysis of human muscle physiology.

Muscle motor unit recruitment during exercise is an important phenomenon and can be inferred noninvasively from surface EMG signals (Sun et al., 2022). All-out exercise is characterized by a progressive decrease in iEMG, showing a decline in motor unit recruitment as the exercise progresses (30% decrease in iEMG at the end of a 5-min period, Figure 3A). Our simulations indicate that this progressive decline in muscle motor unit recruitment observed during all-out exercise contributes only modestly to the loss of force-generating capacity (Figure 3). Although experimental studies have consistently reported substantial reductions in iEMG during maximal efforts (Burnley, 2009; Vanhatalo et al., 2011), maintaining maximal muscle motor unit recruitment throughout the exercise in our model recovered only ∼13% of the total force decline (Figure 3B). This limited recovery suggests that reductions in neural drive alone cannot account for the pronounced force loss and that peripheral fatigue mechanisms play a dominant role. During all-out exercise, rapid accumulation of metabolites, such as H^+^ and P_i_, is known to impair excitation-contraction coupling by reducing calcium release, decreasing myofibrillar calcium sensitivity, and lowering force per cross-bridge (Allen et al., 2008; Fitts, 2008), thereby limiting force output even under maximal effort. The observed reduction in muscle motor unit recruitment may therefore reflect a secondary, protective response to peripheral fatigue mediated by metabolite-sensitive group III/IV afferent feedback rather than its primary cause (Gandevia et al., 1996; Amann and Dempsey, 2008). Indeed, it has been reported that even though both iEMG and work rate decreased during the course of an all-out sprint exercise, the iEMG/work rate ratio continuously increased until reaching a value of ∼4 and then remained unchanged until the end of the exercise (Vanhatalo et al., 2011). Similarly, during all-out knee extension exercise, the quadriceps’ twitch force was shown to decrease at a faster rate than the voluntary activation (Hureau et al., 2022). Based on these observations, force-generating capacity decreased much faster than motor unit recruitment, indicating that the decline in force generation is influenced more by metabolite accumulation than by the decrease in motor unit recruitment during this small muscle mass exercise. In comparison, when subjects performed a constant-power exercise involving submaximal contractions, motor unit recruitment measured by iEMG increased, indicating additional motor units were recruited during the course of the exercise (Vanhatalo et al., 2011; Contessa et al., 2016). In this case, there was an increased muscle motor unit recruitment in order to compensate for the effect of fatigue on force generation (Vanhatalo et al., 2011; Contessa et al., 2016). In fact, our earlier study using the same modeling framework but focusing on a constant-power plantar flexion exercise showed that this increase in motor unit recruitment is crucial for maintaining a constant power output during exercise (Hendry et al., 2025).

Given that alterations in muscle metabolite levels play a significant role in muscle fatigue development during exercise, we investigated the effects of P_i_ and H^+^ on force generation. Our combined experimental and computational approach showed that force production was significantly impacted by P_i_ accumulation (∼30 mM) during an all-out knee extension exercise in humans. Our findings match those reported from *in vitro* experiments on Ca^2+^-activated rabbit and rat fibers, where elevated concentrations of P_i_ (25–30 mM) led to a 5%–19% decrease in force production (Coupland et al., 2001; Debold et al., 2004). Similarly, our analysis indicated that force production was majorly impacted by H^+^ accumulation as well. In fact, other studies reported a 10%–20% decline in force even at pH values of 6.5–6.6 (Westerblad et al., 1997; Woodward and Debold, 2018; Hureau et al., 2022). Thus, our model predictions showed that P_i_ and H^+^ accumulation are two factors contributing to the observed peripheral fatigue development in humans performing an all-out knee extension exercise. In our previous study, we observed a similar behavior when individuals performed a constant-power plantar flexion exercise; however, the effect of H^+^ on force production was marginal compared to P_i_ for that exercise modality (Hendry et al., 2025). One implication of this observation is that the impact of H^+^ and P_i_ accumulation on fatigue development might vary for different athletic activities. As a consequence, in addition to tailored nutritional and training adaptations to reduce muscle fatigue development, we could evaluate whether athletic performance can be improved by increased proton-buffering strategies in the case of “anaerobic” all-out events and by increased energy efficiency and ATP turnover to reduce P_i_ accumulation in more “aerobic” steady-state events.

Strengthening our argument for force production’s differential sensitivity to altered metabolic factors based on exercise routine, our parameter sensitivity analysis revealed that force generation during an all-out knee extension exercise was more sensitive to H^+^ than P_i_, with higher perturbations observed in response to changes in H^+^ than P_i_ (Figures 4, 5). In contrast, we observed the opposite effect when individuals performed a constant-power plantar flexion exercise (Hendry et al., 2025); force generation was more sensitive to P_i_ than H^+^ (see Supplementary Figure S1). In fact, we observed an 84% higher H^+^ accumulation when individuals performed a knee extension exercise (343 nM) compared to a plantar flexion exercise (186 nM). These results suggest that the factors that contribute to muscle fatigue development depend on the exercise modality performed (all-out versus constant-load) and that H^+^ accumulation plays a more significant role than P_i_ accumulation during a high-intensity all-out knee extension exercise. In addition to the differences associated with all-out versus constant-power exercises, several other factors, such as muscle mass (Rossman et al., 2014), fiber type, and contraction mode (isometric versus dynamic), can contribute to the observed differences in the sensitivity of force to metabolic levels. A straightforward comparison of these two exercise types (all-out versus constant-power) from the same muscle type would provide better insight into the underlying causes of this difference in force sensitivity.

In the four-state cross-bridge cycling model, release of P_i_ from the weakly bound state (A_1_) is a prerequisite for the subsequent formation of the strongly bound state (A_2_) (Figure 1A), as proposed in earlier studies (Pate and Cooke, 1989; Kawai et al., 1993; Tewari et al., 2016; Muangkram et al., 2020). As a result, increased concentrations of P_i_ positively modulate the rate of A_1_→P transition, leading to cross-bridge detachment (Supplementary Appendix Equation A5), and negatively modulate the rate of A_1_→A_2_ transition, preventing the formation of a strongly bound state (Supplementary Appendix Equation A6). Our simulations indeed showed that increasing P_i_ concentration increased the fraction of cross-bridges in the P state (Figure 4D), indicating an increase in cross-bridge detachment, as proposed in earlier studies (Linari et al., 2010; Caremani et al., 2013; Debold et al., 2016). Furthermore, experiments based on laser trap assays and mini ensembles of myosin molecules have shown that increasing P_i_ concentration promoted detachment of myosin from actin (Debold et al., 2013). In addition, our model predicted an increased accumulation of cross-bridges in the A_1_ state (Figure 4D) with increasing P_i_, which indicates a possible decreased rate of transition from the weakly bound to the strongly bound state, suggesting potential additional mechanisms through which P_i_ can inhibit force generation. In the case of increasing H^+^ concentrations, our model predicted an increase in the fraction of cross-bridges in the A_1_ state while the fractions of all the other states decreased, which indicates inhibition of the formation of a strongly bound state (A_2_) as a potential mechanism for H^+^-mediated inhibition of force generation in skeletal muscles, as proposed in earlier studies (Sundberg et al., 2018). Studies using laser trap assays on mini-muscle ensembles have generated data that support the possibility of this mechanism of action for H^+^ in inhibiting cross-bridge cycling (Jarvis et al., 2018; Woodward and Debold, 2018). Thus, along with pinpointing the factors causing muscle fatigue, our model was also able to shed light on potential mechanisms through which these factors could inhibit force generation.

## 5 Limitations and future perspectives

### 5.1 Model assumptions and limitations

In addition to directly impacting cross-bridge dynamics, H^+^ and P_i_ have been proposed to impact force generation through mechanisms that modulate cell Ca^2+^ dynamics, including *1*) H^+^-mediated modulation of myofibrillar ATPase activity (Fitts, 2016), *2*) H^+^-mediated regulation of Ca^2+^-troponin interaction (Nelson and Fitts, 2014), *3*) modulation of ATPase activity of Na^+^-K^+^ channels on the sarcolemma (Fitts, 2016) and Ca^2+^ channels in the sarcoplasmic reticulum (SR) membrane (Wolosker et al., 1997), *4*) precipitation of Ca^2+^-P_i_ in the SR (Westerblad and Allen, 1996), and *5*) P_i_-mediated inhibition of ryanodine receptors on the surface of the SR (Duke and Steele, 2001). Incorporating these mechanisms into our model would introduce additional parameters whose estimation would require Ca^2+^ dynamics data. Measuring intracellular Ca^2+^ dynamics in exercising humans is challenging, and these data are not currently available. Therefore, we did not include these mechanisms in the current version of our model. For the same reason, we also did not model the Ca^2+^-mediated activation of muscles; instead, we used iEMG as an indirect measure of muscle motor unit recruitment representing muscle activation. In addition, the accumulation of P_i_ might also lower the free energy change associated with ATP hydrolysis, thereby impacting energetics and potentially leading to a reduction in contractile performance, as hypothesized in the case of cardiac tissues (Wu et al., 2008). In its current form, our model does not account for energetics because ATP levels have been reported to remain largely unaltered in the associated muscles during all-out knee extension exercise (Broxterman et al., 2017). Lastly, although the iEMG signal predominantly arises due to neural activity, it may also be influenced by the metabolite accumulation occurring within the muscle cells as a result of contraction, which the current model does not capture.

Regardless of these limitations, as demonstrated in this study, our model offers a unique framework to integrate information from multiple experimental measures, such as muscle metabolite alterations, force profile, and iEMG, to quantify the potential roles these factors play in muscle force generation and fatigue development. The model as well as the data used for its parameterization capture the average behavior of the muscles under investigation; therefore, the model predictions cannot be generalized to the individual fibers that make up the muscle.

### 5.2 Future perspectives

In muscle cells, the metabolite pools are compartmentalized into different organelles/compartments, such as mitochondria, SR, cytoplasm, etc. Such compartmentalization can be modelled using data from compartment-specific measurement of metabolite pools. Accounting for such segregated metabolite pools, as demonstrated in recent studies (Lopez et al., 2020), would improve the accuracy of the model and allow us to study the effect of energy shuttle reactions on force generation. Incorporating processes such as oxidative phosphorylation and glycolysis in a detailed fashion would also be another avenue to explore, as this would help us better capture the role of these metabolic processes in muscle fatigue development. Another important physiological aspect to explore would be the effect of heterogeneity in the proportions of different motor unit types on fatigue development. This would help in evaluating the extent to which the observations from this study could be generalized across individuals with different proportions of motor unit types. Finally, incorporating additional factors related to the central nervous system could significantly enhance the model’s capability to simulate the experimental data and delineate the potential role of various other key contributing factors in the development of muscle fatigue.

## 6 Conclusion

In this study, we developed a human skeletal muscle computational model that accounts for the actin-myosin cross-bridge cycle and associated metabolic processes to simulate the force generation and metabolite accumulation observed during an all-out knee extension exercise. We parameterized the model using experimental data collected as individuals performed an all-out knee extension exercise, including surface EMG, force output, and muscle metabolite accumulations. Our simulations indicated that the observed progressive decrease in motor unit recruitment during an all-out knee extension exercise contributed minimally to the decline in force generation. Using our combined experimental and computational approach, we identified that accumulation of muscle metabolites, such as P_i_ and H^+^, plays a dominant role in muscle force generation and muscle fatigue development. In particular, our model simulations revealed that muscle force generation is more sensitive to H^+^ than P_i_ during an all-out knee extension exercise. In addition, we identified potential mechanisms in the cross-bridge cycle where these metabolites bind and alter its kinetics during force generation. Our model simulations indicated that P_i_ inhibited cross-bridge cycling by increasing actin-myosin detachment and H^+^ impacted force generation by preventing the formation of a strongly bound cross-bridge state, as proposed in earlier experimental studies.

## Data Availability

The datasets presented in this study can be found in online repositories. The names of the repository/repositories and accession number(s) can be found in the article/Supplementary Material.
