# Supplementary material for: Cross-bridge model-based quantification of muscle metabolite alterations leading to fatigue during all-out knee extension exercise
Source: Front Physiol. 2026 Mar 20;17:1741796. doi: 10.3389/fphys.2026.1741796 (PMC13046550; doi:10.3389/fphys.2026.1741796)
Supplement: Supplementary file 1 [file DataSheet1.docx]

**Cross-bridge model-based quantification of muscle metabolite alterations leading to fatigue during all-out knee extension exercise**

**John I. Hendry^1,2^, Muhammet Enes Erol^3,4^, Gwenael Layec^3,4^, Edward P. Debold^3^,
Anders Wallqvist^1,*^, and Venkat R. Pannala^1,2,*^**

^1^Department of Defense Biotechnology High Performance Computing Software Applications Institute, Defense Health Agency Research & Development, Medical Research and Development Command, Fort Detrick, MD 21702, USA

^2^The Henry M. Jackson Foundation for the Advancement of Military Medicine, Inc., Bethesda, MD 20817, USA

^3^Department of Kinesiology, University of Massachusetts, Amherst, MA 01003, USA

^4^School of Health and Kinesiology, University of Nebraska, Omaha, NE 68182, USA

*Correspondence:

Anders Wallqvist, Ph.D.

Department of Defense Biotechnology High Performance Computing Software Applications Institute

Defense Health Agency Research & Development

Medical Research and Development Command

ATTN: FCMR-TT, 504 Scott Street

Fort Detrick, MD 21702-5012

Email: sven.a.wallqvist.civ@health.mil; Phone: (301) 619-1989

Venkat Pannala, Ph.D.

Email: vpannala@bhsai.org; Phone: (301) 619-1978

Supplementary Material

# Appendices

## Appendix A: Modeling cross-bridge cycling and force generation

We used *P*(*t*), *p*_1_(*t,s*), *p*_2_(*t,s*)*,* and *p*_3_(*t,s*) to denote the state probabilities of the cross-bridge states (P, A_1_, A_2_, and A_3_) illustrated in **Figure 1A**, where *s* represents the strain on the attached states (A_1_, A_2_, and A_3_) and *t* denotes the time in seconds.

We used the following equations from Tewari et al. (Tewari et al., 2016) to model the cross-bridge cycling kinetics, the associated changes in cross-bridge state probabilities, and the metabolite-mediated modulation of cross-bridge cycling kinetics.

| \|  \| $\frac{\text{d}\text{P}}{\text{d}\text{t}}\text{ }\text{=}{\tilde{\text{k}}}_{\text{d}}\text{p}_{\text{1}}\left( \text{t}\text{,}\text{s} \right) \text{-}\text{ }\text{k}_{\text{a}}\text{P}\left( \text{t} \right)\text{ }\text{+ }{\tilde{\text{k}}}_{\text{3}}\text{e}^{\text{α}_{\text{3}}{\text{(}\text{s}\text{+}\text{s}_{\text{3}}\text{)}}^{\text{2}}}\text{p}_{\text{3}}\left( \text{t}\text{,}\text{s} \right)$ \| (A1) \| \| --- \| --- \| --- \| \|  \| $\frac{\text{∂}\text{p}_{\text{1}}}{\text{∂}\text{t}}\text{ }\text{+}\text{ }\frac{\text{d}\text{L}}{\text{d}\text{t}}\frac{\text{∂}\text{p}_{\text{1}}}{\text{∂}\text{s}}\text{ }\text{=}{\text{ }\text{k}}_{\text{a}}\text{δ}\left( \text{s} \right)\text{P}\left( \text{t} \right) -\text{ }{\tilde{\text{k}}}_{\text{d}}\text{p}_{\text{1}}-\text{ }{\tilde{\text{k}}}_{\text{1}}\text{e}^{\text{-α}_{\text{1}}\text{s}}\text{p}_{\text{1}}\text{+}\text{ }{\tilde{\text{k}}}_{\text{-1}}\text{e}^{\text{+α}_{\text{1}}\text{s}}\text{p}_{\text{2}}$ \| (A2) \| \|  \| $\frac{\text{∂}\text{p}_{\text{2}}}{\text{∂}\text{t}}\text{ }\text{+}\text{ }\frac{\text{d}\text{L}}{\text{d}\text{t}}\frac{\text{∂}\text{p}_{\text{2}}}{\text{∂}\text{s}}\text{=}\text{ }{\tilde{\text{k}}}_{\text{1}}\text{e}^{\text{-α}_{\text{1}}\text{s}}\text{p}_{\text{1}} -\text{ }\text{k}_{\text{-1}}\text{e}^{\text{+α}_{\text{1}}\text{s}}\text{p}_{\text{2}} -\text{ }\text{k}_{\text{2}}\text{e}^{\text{-α}_{\text{2}}\text{s}}\text{p}_{\text{2}}\text{+}\text{ }{\tilde{\text{k}}}_{\text{-2}}\text{p}_{\text{3}}$ \| (A3) \| \|  \| $\frac{\text{∂}\text{p}_{\text{3}}}{\text{∂}\text{t}}\text{ }\text{+}\text{ }\frac{\text{d}\text{L}}{\text{d}\text{t}}\frac{\text{∂}\text{p}_{\text{3}}}{\text{∂}\text{s}}\text{ }\text{=}{\text{ }\text{k}}_{\text{2}}\text{e}^{\text{-α}_{\text{2}}\text{s}}\text{p}_{\text{2}} -{\tilde{\text{ }\text{k}}}_{\text{-2}}\text{p}_{\text{3}} -\text{ }{\tilde{\text{k}}}_{\text{3}}\text{e}^{\text{α}_{\text{3}}{\text{(}\text{s}\text{+}\text{s}_{\text{3}}\text{)}}^{\text{2}}}\text{p}_{\text{3}}$ \| (A4) \| \|  \| ${\tilde{\text{k}}}_{\text{d}}\text{ }\text{=}\text{ }\text{k}_{\text{d}}\frac{{\text{[P}_{\text{i}}\text{]}}/{\text{K}_{\text{Pi}}}}{\text{1}\text{ }\text{+}\text{ }{\text{[P}_{\text{i}}\text{]}}/{\text{K}_{\text{Pi}}}}$ \| (A5) \| \|  \| ${\tilde{\text{k}}}_{\text{1}}\text{ }\text{=}\text{ }\text{k}_{\text{1}}\frac{\text{1}}{\text{1}\text{ }\text{+}\text{ }{\text{[P}_{\text{i}}\text{]}}/{\text{K}_{\text{Pi}}}}$ \| (A6) \| \|  \| ${\tilde{\text{k}}}_{\text{-1}}\text{ = }\text{k}_{\text{-1}}\frac{\left[ \text{H}^{\text{+}} \right]/{\text{K}_{\text{H+}}}}{\text{1 + }\left[ \text{H}^{\text{+}} \right]/{\text{K}_{\text{H+}}}}$ \| (A7) \| \|  \| ${\tilde{\text{k}}}_{\text{2}}\text{ }\text{=}\text{ }\text{k}_{\text{2}}\frac{\text{1}}{\text{1}\text{ }\text{+}\left[ \text{H}^{\text{+}} \right]/{\text{K}_{\text{H+}}}}$ \| (A8) \| \|  \| ${\tilde{\text{k}}}_{\text{-2}\text{ }}\text{=}\text{ }\text{k}_{\text{-2}}\frac{\text{[ADP]}/{\text{K}_{\text{ADP}}}}{\text{1}\text{ }\text{+}\text{ }\text{[ADP]}/{\text{K}_{\text{ADP}}}\text{ }\text{+}\text{[ATP]}/{\text{K}_{\text{ATP}}}\text{+}\text{ }\text{(}\left[ \text{ADP} \right]/{\text{K}_{\text{ADP}}}\text{)}}$ \| (A9) \| \|  \| ${\tilde{\text{k}}}_{\text{3}\text{ }}\text{=}\text{ }\text{k}_{\text{3}}\frac{\text{[ATP]}/{\text{K}_{\text{ATP}}}}{\text{1}\text{ }\text{+}\text{ }\text{[ADP]}/{\text{K}_{\text{ADP}}}\text{+}\text{[ATP]}/{\text{K}_{\text{ATP}}}\text{+}\text{ }\text{(}\left[ \text{ADP} \right]/{\text{K}_{\text{ADP}}}\text{)}}$ \| (A10) \| |  |  |
| --- | --- | --- | --- | --- | --- | --- | --- | --- | --- | --- | --- | --- | --- | --- | --- | --- | --- | --- | --- | --- | --- | --- | --- | --- | --- | --- | --- | --- | --- | --- | --- | --- |

where *L* represents the sarcomere length, d*L/*d*t* denotes the sarcomere shortening velocity, and [P_i_], [ADP], [ATP], and [H^+^] represent the concentrations of P_i_, ADP, ATP, and H^+^ (in mM), respectively. Since we simulate isometric contractions in this study, the sarcomere shortening velocity (d*L/*d*t*) was set to zero. For a description of all the parameters used in Equations A1−A10, please see **Table 1** in the main text.

The fraction of total cross-bridges that exist in one of the three attached states *i* is related to its state probabilities using the following relation:

|  | $\hat{\text{p}_{\text{i}}}\left( \text{t} \right)\text{ =}\int_{\text{-∞}}^{\text{∞}} \text{p}_{\text{i}}\text{(}\text{t}\text{,}\text{s}\text{)d}\text{s}$ | (A11) |
| --- | --- | --- |

The model also assumes that the sum of the fractions of all four states equals the initial permissible state fraction (P) at any given time, as given by the following equation:

|  | $\text{P}\left( \text{t} \right)\text{ }\text{+}\int_{\text{-∞}}^{\text{+∞}} \text{p}_{\text{1}}\left( \text{t}\text{,}\text{s} \right)\text{d}\text{s}\text{ }\text{+}\int_{\text{-∞}}^{\text{+∞}} \text{p}_{\text{2}}\text{(}\text{t}\text{,}\text{s}\text{)d}\text{s}\text{ }\text{+ }\int_{\text{-∞}}^{\text{+∞}} \text{p}_{\text{3}}\left( \text{t}\text{,}\text{s} \right)\text{d}\text{s}\text{ }\text{=}\text{ }\text{P}\left( t_{0} \right)$ | (A12) |
| --- | --- | --- |

The mean strain of the cross-bridges in one of the attached states *i* is given by $\int_{\text{-∞}}^{\text{+∞}} \text{sp}_{\text{i}}\left( \text{t}\text{,}\text{s} \right)\text{d}\text{s}\text{/}\hat{\text{p}_{\text{i}}}\left( \text{t} \right)$. We calculated the force F_XB_ generated by the cross-bridge cycle as the sum of the force generated due to cross-bridge cycling and the force generated due to the strain in the strongly attached states A_2_ and A_3_, as indicated by the following equation:

|  | $\text{F}_{\text{XB}\text{ }}\text{=}\text{ }\text{k}_{\text{stiff,1}}\left( \int_{\text{-∞}}^{\text{+∞}} \text{sp}_{\text{2}}\left( \text{t}\text{,}\text{s} \right)\text{ds}\text{+}\int_{\text{-∞}}^{\text{+∞}} \text{sp}_{\text{3}}\left( \text{t}\text{,}\text{s} \right)\text{d}\text{s} \right)\text{+}\text{ }\text{k}_{\text{stiff,2}}\text{∆}_{\text{r}}\int_{\text{-∞}}^{\text{+∞}} \text{p}_{\text{3}}\text{(}\text{t}\text{,}\text{s}\text{)d}\text{s}$ | (A13) |
| --- | --- | --- |

where *k*_stiff,1_ represents the stiffness constant for frictional forces arising due to the attached states, *k*_stiff,2_ denotes the stiffness constant of ratcheted cross-bridges, and $\text{∆}_{\text{r}}$ represents the size of the power stroke. We calculated the total force F_total_ generated by the muscle as the sum of the force generated by the actin-myosin contraction and the elastic force generated by the cartilage, referred to as the PEE.

|  | $\text{F}_{\text{total}\text{ }}\text{=}\text{ }\text{F}_{\text{XB}}\text{ }\text{+}\text{ }\text{F}_{\text{PEE}}$ | (A14) |
| --- | --- | --- |

We calculated the force contribution from PEE using the method of Rockenfeller et al. (2020).

## Appendix B: Modeling metabolite alterations in skeletal muscles

**Table B1** summarizes the different metabolic processes, their stoichiometric equations, and their rate expressions as used in the model. Based on the stoichiometry and the corresponding rate expressions, we formulated the equations governing the alterations in the various metabolites using mass balance principles as follows:

|  | $\frac{\text{d}\text{[P}_{\text{i}}\text{]}}{\text{d}\text{t}}\text{ }\text{=}{\tilde{\text{ }\text{k}}}_{\text{3}}\text{e}^{\text{α}_{\text{3}}{\text{(}\text{s}\text{+}\text{s}_{\text{3}}\text{)}}^{\text{2}}}\text{p}_{\text{3}}-\text{k}_{\text{Gly}}\text{[ADP]}\text{[P}_{\text{i}}\text{]}\text{ }\text{-}{\text{ }\text{k}}_{\text{Pi,dil}}\text{[P}_{\text{i}}\text{]}$ | (B1) |
| --- | --- | --- |
|  | $\frac{\text{d}\left[ \text{H}^{\text{+}} \right]}{\text{d}\text{t}}\text{ }\text{=}\text{ }\text{0.6*}{\tilde{\text{k}}}_{\text{3}}\text{e}^{\text{α}_{\text{3}}\left( \text{s}\text{+}\text{s}_{\text{3}} \right)^{\text{2}}}\text{p}_{\text{3}}\text{+}\text{ }\text{k}_{\text{Gly}}\text{[ADP]}\text{[P}_{\text{i}}\text{]}\text{ }\text{-}\text{ }\text{k}_{\text{CKf}}\text{[PCr][ADP]}$  $\text{+ }\text{k}_{\text{CKr}}\text{(}\left[ \text{PCr} \right]_{\text{0}} -\text{[PCr])[ADP]}\text{ }\text{+}\text{ }\text{γ}\text{ΔpH}$ | (B2) |
|  | $\frac{\text{d}\left[ \text{ADP} \right]}{\text{d}\text{t}}\text{ }\text{=}\text{ }{\tilde{\text{k}}}_{\text{3}}\text{e}^{\text{α}_{\text{3}}{\text{(}\text{s}\text{+}\text{s}_{\text{3}}\text{)}}^{\text{2}}}\text{p}_{\text{3}} \text{–}{\text{ }\text{k}}_{\text{Gly}}\text{[ADP]}\text{[P}_{\text{i}}\text{]}\text{–}\text{k}_{\text{CKf}}\text{[PCr][ADP]}$  $\text{+}\text{ }\text{k}_{\text{CKr}}\text{(}\left[ \text{PCr} \right]_{\text{0}}\text{ }\text{–}\text{ }\text{[PCr])[ADP]}\text{ }\text{–}\text{ }\text{k}_{\text{adk}}\text{[ADP][ADP]}\text{ }$ | (B3) |
|  | $\frac{\text{d}\left[ \text{PCr} \right]}{\text{d}\text{t}}\text{=}\text{ }\text{–}\text{k}_{\text{CKf}}\text{[PCr][ADP]}\text{ }\text{+}\text{ }\text{k}_{\text{CKr}}\text{(}\left[ \text{PCr} \right]_{\text{0}} -\text{[PCr])[ADP]}$ | (B4) |
|  | $\frac{\text{d}\left[ \text{ADP} \right]}{\text{d}\text{t}}\text{=}\text{ }\text{-}\frac{\text{d}\left[ \text{ATP} \right]}{\text{d}\text{t}}$ | (B5) |

For a complete description of all the parameters in the above equations, please see **Table 1** in the main text.

**Table B1.** Metabolic processes, their stoichiometric chemical equations, and their rate expressions used in the model.

| **Number** | **Reaction/pathway** | **Chemical equation** | **Rate formulation** |
| --- | --- | --- | --- |
| 1 | ATP hydrolysis | ^*^$\text{ATP}\text{ }\text{+}\text{ }\text{H}_{\text{2}}\text{O}\text{ }\text{→}\text{ }\text{ADP}\text{ }\text{+}\text{ }\text{P}_{\text{i}}\text{ }\text{+}\text{ }\text{0.6}\text{H}^{\text{+}}$ | ${\tilde{\text{k}}}_{\text{3}}\text{e}^{\text{α}_{\text{3}}{\text{(}\text{s}\text{+}\text{s}_{\text{3}}\text{)}}^{\text{2}}}\text{p}_{\text{3}}$ |
| 2 | Creatine kinase (ATP buffering) | $\text{PCr + ADP + }\text{H}^{\text{+}}\text{ → ATP + Cr}$ | $\text{k}_{\text{CKf}}\text{[PCr][ADP]}$ |
| 3 | Creatine kinase (PCr regenerating) | $\text{ATP} \text{+ Cr → PCr + ADP} \text{+}\text{ H}^{\text{+}}$ | $\text{k}_{\text{CKr}}\text{(}\left[ \text{PCr} \right]_{\text{0}}-\text{[PCr])[ADP]}$ |
| 4 | ATP generation processes | $\text{A}\text{DP + }\text{P}_{\text{i}}\text{ }\text{→}\text{ ATP + }\text{H}_{\text{2}}\text{O}\text{ + }\text{ H}^{\text{+}}\text{ }$ | $\text{k}_{\text{Gly}}\text{[ADP]}\text{[P}_{\text{i}}\text{]}$ |
| 5 | Adenylate kinase | $\text{ADP + ADP → ATP + AMP}$ | $\text{k}_{\text{adk}}\text{[ADP][ADP]}$ |

Cr, creatine; PCr, phosphocreatine. ^*^We set the stoichiometric coefficient of H^+^ to 0.6 based on the ratio at which H_2_PO_4_^-^ and HPO_4_^2-^ constitute P_i_ at physiological pH (Kushmerick, 2011).

# Supplementary Figure


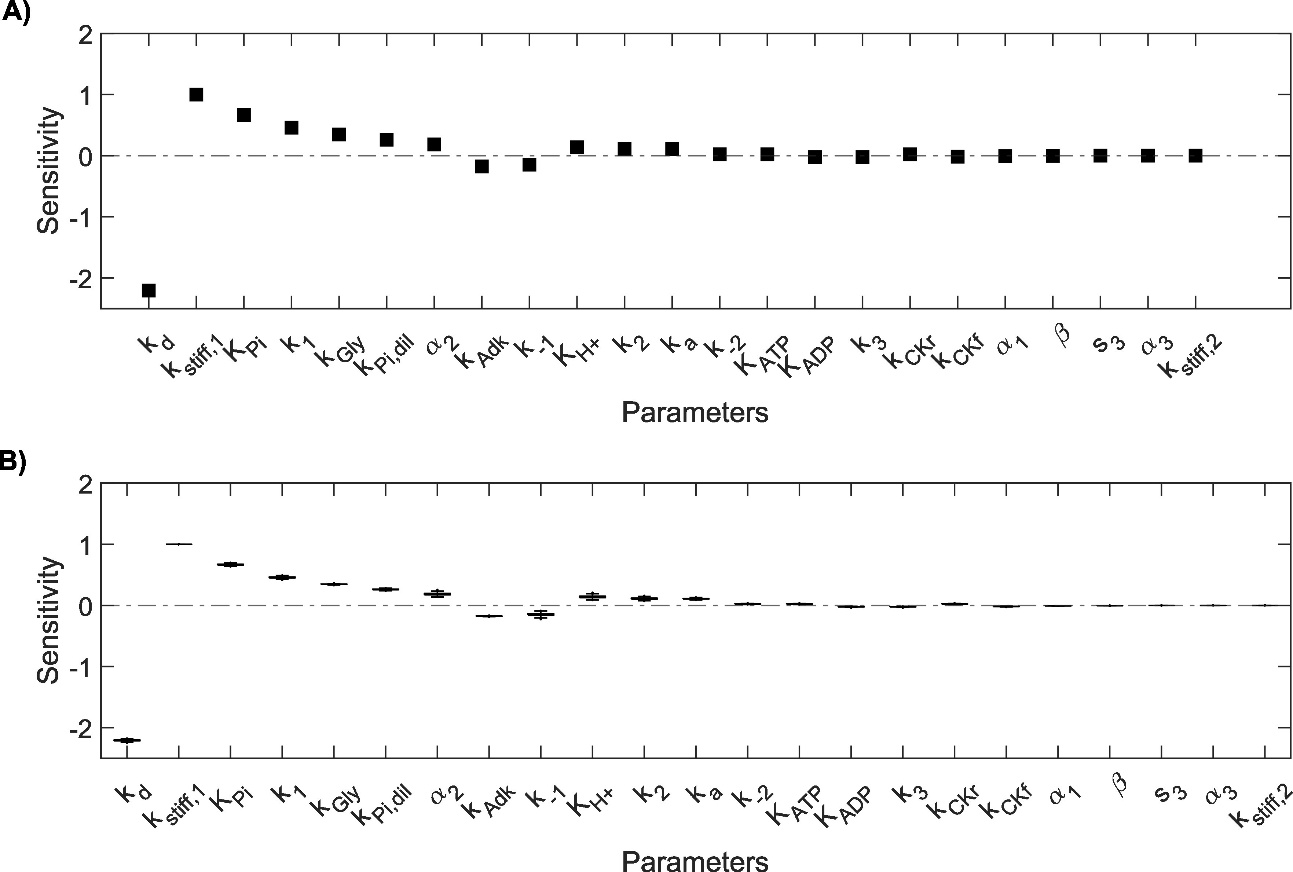


**Figure S1.** Parameter sensitivity analysis of the cross-bridge model for a dynamic plantar flexion exercise. A) Plot of the local sensitivities calculated for all 23 parameters in the model. B) Box plot showing the global sensitivities for the parameters. To perform the global sensitivity analysis, we pooled 10,000 parameter sets by sampling the 10% neighborhood of the optimal parameter set. We then calculated the force sensitivities for each of the 10,000 sampled parameter sets and summarized the values in a box plot.

# References

Kushmerick, M.J. (2011). "Energetics of muscle contraction," in *Comprehensive Physiology*. (Hoboken, NJ.: Wiley), 189–236.

Rockenfeller, R., Gunther, M., Stutzig, N., Haeufle, D.F.B., Siebert, T., Schmitt, S., et al. (2020). Exhaustion of skeletal muscle fibers within seconds: incorporating phosphate kinetics into a Hill-type model. *Front Physiol* 11**,** 306. doi: 10.3389/fphys.2020.00306.

Tewari, S.G., Bugenhagen, S.M., Palmer, B.M., and Beard, D.A. (2016). Dynamics of cross-bridge cycling, ATP hydrolysis, force generation, and deformation in cardiac muscle. *J Mol Cell Cardiol* 96**,** 11–25. doi: 10.1016/j.yjmcc.2015.02.006.
